# Incidental Diagnosis of Metastatic Colorectal Cancer in a Habitual Hookah Smoker: A Case Report Emphasizing Early Detection and Lifestyle Risks

**DOI:** 10.7759/cureus.58204

**Published:** 2024-04-13

**Authors:** Omar B Banamah, Renad A Sagim, Abdullah A Al Qurashi

**Affiliations:** 1 Family Medicine, King Abdulaziz Medical City, Jeddah, SAU; 2 College of Medicine, King Saud Bin Abdulaziz University for Health Sciences, Jeddah, SAU

**Keywords:** elevated liver-associated enzymes, early detection of cancer, family medicine, colorectal metastasis, incidental diagnosis, hookah smoking, colorectal cancer

## Abstract

Colorectal cancer (CRC) is a major health concern and a significant contributor to global oncological mortality, influenced by genetic predisposition and lifestyle factors. Hookah smoking, prevalent in the Middle East, has been associated with an increased risk of various cancers, including CRC. This case report discusses the incidental discovery of metastatic CRC in a 42-year-old habitual hookah smoker, shedding light on the potential association between hookah use and CRC pathogenesis. Additionally, it addresses the diagnostic complexities posed by the asymptomatic nature of CRC, often identified through non-specific indicators such as abnormal liver enzymes. Furthermore, the case illustrates the crucial role of family medicine in detecting diseases, highlights the significance of multidisciplinary care in managing advanced CRC, and emphasizes the importance of public health initiatives to raise awareness about the risks of hookah smoking and promote regular health screenings in at-risk populations.

## Introduction

Colorectal cancer (CRC) is a leading cause of oncological mortality worldwide, with its etiology linked to both genetic and lifestyle factors [1]. CRC may not initially present with symptoms, but if it does, individuals may experience abdominal pain, diarrhea, constipation, or blood in the stool. In some cases, signs of liver involvement, such as a palpable liver on examination, jaundice, or abnormal liver enzyme levels, may indicate the spread of the cancer to the liver [2,3]. Notably, the consumption of hookah, or waterpipe tobacco, a cultural practice deeply rooted in Middle Eastern societies, including Saudi Arabia, has been implicated in the elevated risk of various malignancies, including CRC [4]. Recent epidemiological studies have begun to shed light on the carcinogenic potential of hookah smoking, suggesting a significant association with colorectal carcinogenesis [5,6]. This case report details the incidental diagnosis of metastatic CRC in a 42-year-old male, a habitual hookah smoker, thereby exploring the potential correlation between hookah use and CRC pathogenesis. It also highlights the diagnostic challenges posed by the asymptomatic nature of CRC, often discovered through non-specific indicators such as abnormal liver enzymes. Furthermore, it discusses the implications of such findings in clinical practice.

## Case presentation

A 42-year-old male, an active smoker with a notable history of hookah use for 22 years, presented to the family medicine clinic for evaluation due to persistent iron deficiency anemia and elevated liver enzymes, with hepatitis ruled out. He also had poorly controlled diabetes mellitus managed on metformin and was newly diagnosed with dyslipidemia. His medical history included right inguinal hernia repair and left hydrocele surgery. Notably, the patient exhibited no significant gastrointestinal symptoms such as nausea, vomiting, diarrhea, or abdominal pain, which underscores the incidental nature of the cancer discovery. Additionally, no family history of colorectal or related cancers warrants earlier screening.

Laboratory findings revealed hemoglobin A1C levels indicative of poorly controlled diabetes, elevated gamma-glutamyl transferase (GGT), and other liver function test abnormalities. Table 1 presents the patient’s laboratory parameters at first presentation.

**Table 1:** The patient’s laboratory parameters upon initial presentation.

Lab parameters	Patient's result	Reference interval
At first presentation:		
Hemoglobin A1C	8%	3.9-6.1%
Alanine transaminase (ALT)	17 U/L	7-44 U/L
Aspartate aminotransferase (AST)	20 IU/L	5-34 IU/L
Alkaline phosphatase (ALP)	148 U/L	39-114 U/L
Gamma-glutamyl transpeptidase (GGT)	103 IU/L	11-68 IU/L

**Figure 1:**
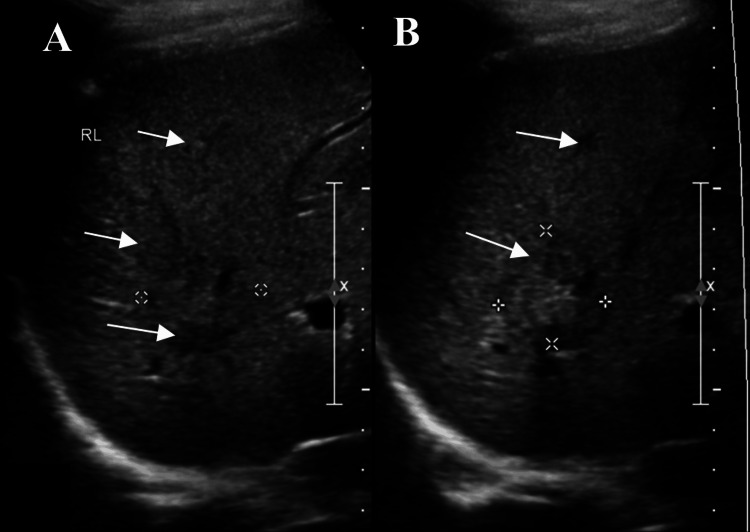
Ultrasound of the abdomen. Ultrasound images of the abdomen demonstrate multiple hepatic masses that require further assessment.

After that, an abdominal ultrasound was performed, which revealed incidental findings of multiple hepatic masses. Further imaging was required to confirm the findings (Figure 1).

**Figure 2:**
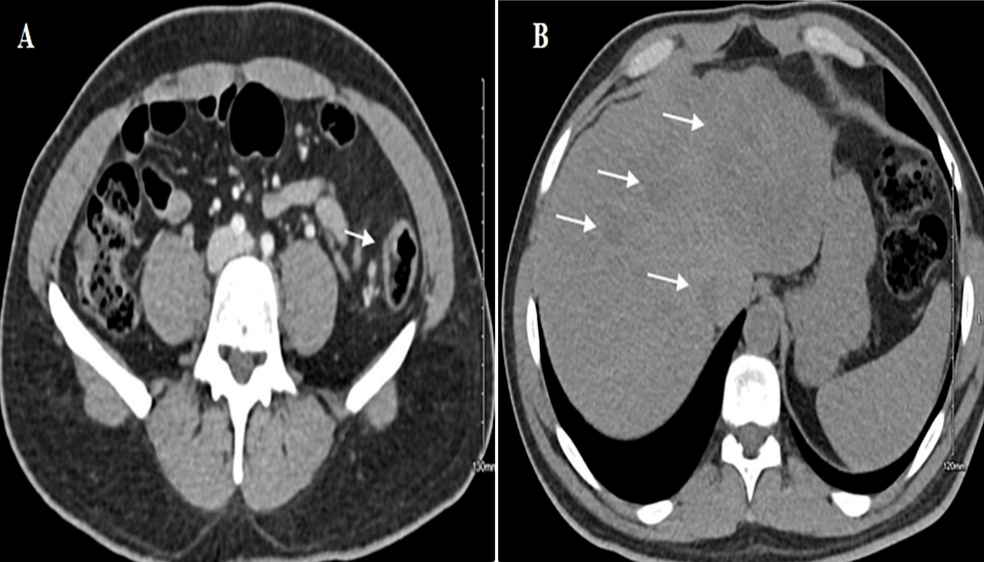
Enhanced axial CT of the abdomen and pelvis. Contrast-enhanced axial images of the abdomen and pelvis show (A) a nonobstructive colonic sigmoid mass with locoregional lymphadenopathy. In addition, (B) multiple innumerable hepatic lesions highly suspicious for metastasis are noted.

Subsequent abdomen and pelvis CT unveiled a nonobstructive colonic sigmoid mass with locoregional lymphadenopathy and multiple innumerable hepatic lesions highly suspicious for metastasis (Figure 2).

**Figure 3:**
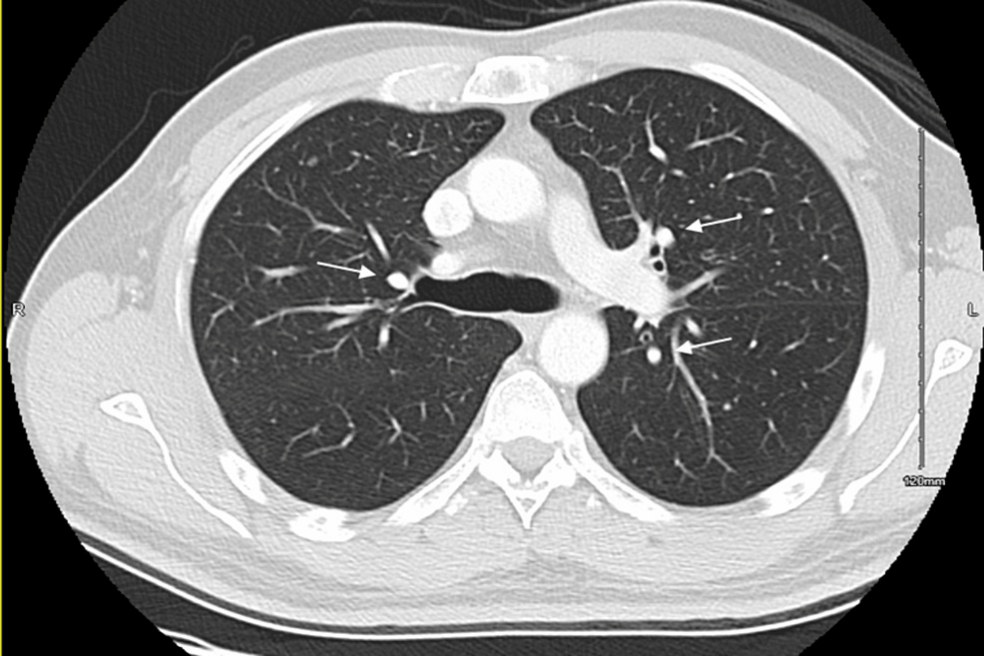
Contrast-enhanced axial CT of the chest. Contrast-enhanced axial image of the chest demonstrates a dominant nodule in the right upper lobe, mostly metastasis. Other nodule characteristics are not suspicious, and they could be inflammatory nodules.

A colonoscopy confirmed a moderately differentiated adenocarcinoma of the descending colon, approximately 55 cm from the anal verge. Additionally, a chest CT scan provided additional detail on the extent of the disease, revealing the involvement of the patient's lungs (Figure 3).

**Figure 4:**
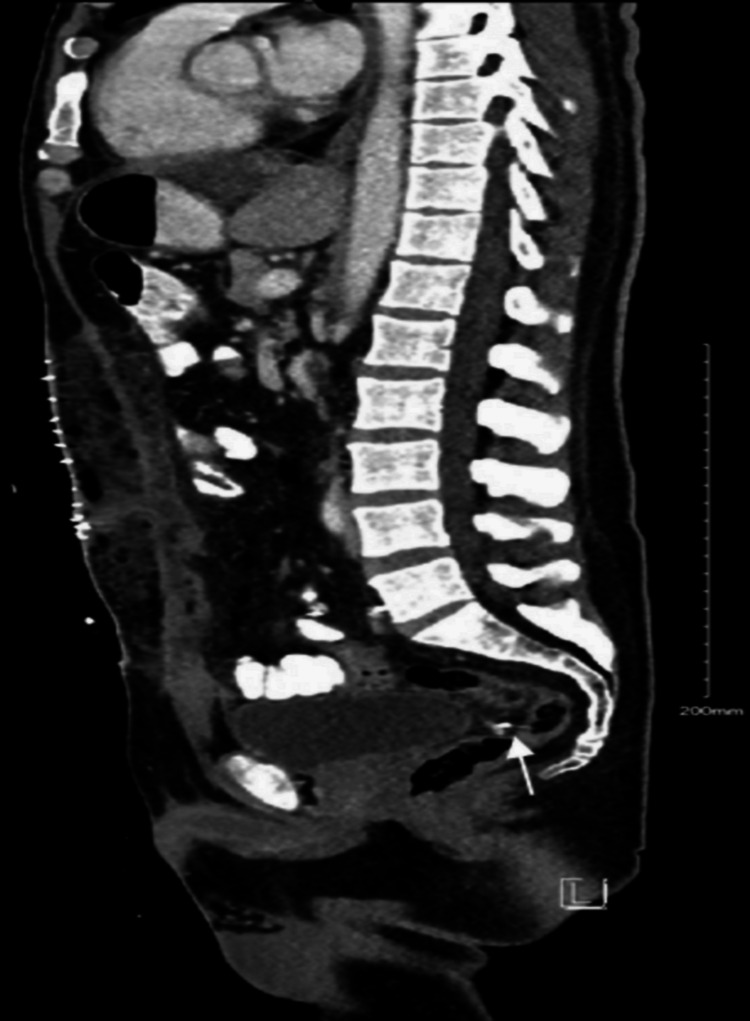
Enhanced sagittal CT scan of the abdomen and pelvis. An enhanced CT scan of the abdomen and pelvis revealed the patient to have alleviated large bowel obstruction following Hartmann’s procedure.

This finding complements the previously identified liver metastasis in the abdominal CT, accentuating the asymptomatic progression of the patient's colorectal cancer. Surgical intervention via a Hartmann’s procedure was performed to address a large bowel obstruction (Figure 4).

The postoperative course was unremarkable, allowing the patient to continue his life with minimal disruption. The postoperative pathology report showed the presence of residual adenocarcinoma post-neoadjuvant therapy. The patient's treatment regimen included cycles of FOLFOX (folinic acid, fluorouracil, and oxaliplatin) + panitumumab, leading to an interval reduction in hepatic metastasis and no evidence of local recurrence.

The patient remained hemodynamically stable throughout his clinical journey, tolerated oral intake well, and experienced no gastrointestinal distress. This case was meticulously discussed in a multidisciplinary tumor board, emphasizing the importance of a coordinated approach in managing metastatic CRC, especially in patients with comorbid conditions and those discovered incidentally through non-specific symptoms or routine evaluations.

This case underscores the critical role of multidisciplinary care in optimizing outcomes for patients with advanced CRC. It highlights the potential etiological link between hookah smoking and the development of CRC. The incidental discovery of metastatic CRC via elevated liver enzymes in this patient illuminates the complex interplay between lifestyle factors and cancer risk, reinforcing the need for awareness and early diagnostic evaluations in at-risk populations.

## Discussion

This case report details the incidental diagnosis of metastatic CRC in a 42-year-old male habitual hookah smoker, emphasizing the essential roles of family medicine in shedding light on the critical need for early detection and tailored screening strategies within this population. Despite the asymptomatic nature of CRC in its early stages, routine screenings such as colonoscopies or fecal occult blood tests (FOBT) can significantly increase the chances of detecting CRC at a more treatable stage [7]. The potential contributory role of hookah smoking in CRC etiology highlighted by this case underscores the importance of comprehensive approaches in managing and preventing CRC.

The cornerstone of family medicine lies in its holistic approach to patient care, combining preventive health measures with managing acute and chronic conditions [8]. This case illustrates family medicine practitioners' essential role in the early detection of CRC by assessing non-specific symptoms and laboratory anomalies. The initial investigation of causes for the patient's persistent iron deficiency anemia and unexplained liver enzyme elevations demonstrates the comprehensive care model integral to family medicine. This model facilitates the early detection of diseases like CRC, which may otherwise remain undetected until advanced stages.

Additionally, it is crucial to address the missed opportunities for early detection and screening specific to this patient population. The patient's history of prolonged hookah smoking serves as a critical reminder of the lifestyle factors contributing to cancer risk. Hookah, often misconceived as a safer alternative to cigarette smoking, contains a variety of carcinogens [9]. The case highlights the urgent need for increased awareness and educational efforts regarding the risks of hookah smoking, particularly in regions where its use is culturally embedded. Advocacy for smoking cessation should be a priority in the preventive health strategies employed by family medicine practitioners, aiming to reduce the well-documented risks associated with tobacco use in all forms [10].

The incidental finding of metastatic CRC via elevated liver enzymes in this patient (elevated alkaline phosphatase and GGT) reports the potential for early disease detection through attentive clinical evaluation. Such findings, while often overlooked, may be caused by underlying malignancies. This case highlights the necessity for an algorithm that promptly addresses unexpected lab results, particularly when risk factors such as hookah smoking are involved. By promptly investigating these abnormalities, we can identify potential issues early, significantly improving patient outcomes.

## Conclusions

In conclusion, this case report contributes to the growing body of literature on CRC and its association with hookah smoking. It also emphasizes the significance of early detection and comprehensive care for advanced CRC cases. In addition, it highlights the crucial role of family medicine in detecting CRC through incidental findings and reinforcing the importance of considering lifestyle factors in patient evaluations. Also, it serves as a reminder of the critical need for public health initiatives focused on educating the community about the risks of smoking and the benefits of regular medical check-ups. Through such efforts, we can hope to improve early cancer detection rates and ultimately save lives.
